# Explainable hybrid machine learning model for predicting stunting and identifying key risk factors among Ethiopian children under five

**DOI:** 10.1038/s41598-026-46417-w

**Published:** 2026-04-09

**Authors:** Tadele Kassahun Wudu, Abraham Alamirew Endalew, Aster Addisu Dires

**Affiliations:** 1 Statistics, Debark University, Debark, Ethiopia; 2 Computer Science, Debark University, Debark, Ethiopia

**Keywords:** Hybrid, Stunting, Childhood stunting, Risk factor of stunting, Computational biology and bioinformatics, Diseases, Health care, Mathematics and computing, Medical research, Risk factors

## Abstract

Childhood stunting, caused by poor nutrition, infections, and inadequate stimulation, impairs growth and development. This leads to reduced cognitive function and academic performance, increased risk of illness and death due to weakened immunity, lower economic productivity, and higher susceptibility to chronic diseases in adulthood. Childhood stunting is a major issue in low-income countries like Ethiopia. Children’s stunting prevalence in Ethiopia is 35.4%, highlighting the need for interventions. The World Health Organization (WHO) classifies stunting as normal, moderate, and severe. The study aims to develop an advanced explainable hybrid predictive model by combining two top-performing algorithms, Extra Tree (ensemble learning) and Multilayer Perceptron (deep learning), to leverage their complementary strengths. This approach enhances accuracy and reveals the inner workings of AI decision-making. The central goal of this study is to move beyond black box models by using post-hoc explainability methods like Local Interpretable Model-agnostic Explanations. This is intended to reveal the inner workings of the artificial intelligence decision-making and identify the key risk factors associated with stunting to build trust for public health policy design. In this study, data from the 2019 Ethiopian Demographic and Health Survey dataset were used for the experiment. The data was preprocessed to ensure high quality for analysis and to build a model predicting childhood stunting. This study employed an experimental research methodology to conduct five experiments, involving 11,121 instances and 17 features. While we developed the model using a balanced dataset, we evaluated it on the original dataset to avoid inflating performance metrics. The experiments showed that the hybrid model constructed from Extra Tree and Multilayer Perceptron performed better than others, achieving an accuracy of 94%. The hybrid model identified age_of_child, Region, Birth_Interval, and number of children under five in the household as the most influential predictors of stunting status in this dataset. It is essential to note that these are predictive risk factors, representing strong statistical associations within the cross-sectional data, and should not be interpreted as definitive causal drivers of stunting.

## Introduction

Childhood stunting refers to impaired growth and development experienced by children due to poor nutrition, repeated infections, and inadequate psychosocial stimulation^1^. Stunting, according to the World Health Organization (WHO), is a height-for-age Z-score (HAZ) below − 2 standard deviations, reflecting chronic undernutrition. Stunting hinders cognitive development and academic achievement, elevates illness and mortality risks due to compromised immunity, diminishes economic productivity, and increases susceptibility to chronic adult diseases like diabetes and obesity^2^. In 2024, 23.2% or 150.2 million children under five were stunted, mainly in low- and middle-income countries, particularly in Asia and Africa, with Sub-Saharan Africa seeing an increase. The global prevalence of stunting declined from 26.4% in 2012 to 23.2% in 2024, but the target of 14% by 2030 is not on track to be met^3^. Childhood stunting is a major public health problem in Ethiopia, contributing to child mortality and impairing long-term growth and development^4^. Prevention and intervention strategies for stunting focus on improving nutrition, access to clean water and sanitation, and healthcare^5^. Good maternal nutrition before and during pregnancy, along with exclusive breastfeeding for six months and nutritious complementary foods, is vital. Stunting in mothers can lead to stunting in their children, creating an intergenerational cycle. For example, a mother who was malnourished during her own childhood is more likely to have a stunted child. Therefore, comprehensive and early interventions are essential to break this cycle and improve long-term health outcomes. Childhood stunting significantly hinders economic growth and productivity through various pathways, including reduced human capital, increased healthcare costs, and decreased economic output^6^. Investing in nutrition and stunting reduction programs is therefore crucial for fostering long-term economic development. Effective stunting reduction requires combined nutrition-specific and nutrition-sensitive interventions^6^. Traditional methods of identifying at-risk children often struggle with the complexity and volume of sociodemographic and health data involved in stunting, requiring more sophisticated and data-driven approaches^7^. This challenge motivates the use of Machine Learning (ML), a subset of Artificial Intelligence (AI) that develops algorithms to learn and predict from large datasets without explicit programming. ML models, particularly powerful ensemble and deep learning algorithms, can harness the complex relationships within comprehensive datasets, such as the Ethiopian Demographic and Health Survey (EDHS), to build advanced predictive models for stunting status. The aim is to move beyond mere prevalence tracking to proactive, accurate identification of children who are severely or moderately stunted. ML models like Random Forest and XGBoost lack interpretability, limiting their use in public health for policy design and resource allocation. Explainable Artificial Intelligence (XAI) is crucial to clarify predictions and build trust by revealing key factors associated with stunting. While these insights do not establish causal relationships due to the cross-sectional nature of the data, they can assist in identifying high-risk groups and informing the design of targeted public health interventions. In this study, we utilized an explainable hybrid model to harness the power of two top-performing ensemble machine learning and deep learning models to build an advanced predictive model.

Machine learning is a subset of artificial intelligence (AI) focused on developing algorithms for computers to learn and predict without explicit programming^8^. It uses statistical techniques to analyze data and identify patterns. Key points in machine learning include learning from data, training on large datasets, and developing models^9^. Machine learning techniques include supervised, unsupervised, Semi-supervised, and reinforcement learning^10^. Supervised learning maps input to output with correct values provided by a supervisor^11^. There are two main types of supervised learning classification and regression. Ensemble learning is grouped under supervised machine learning^11^. The main principle behind ensemble learning is weak learners together to form a strong learner for improved prediction accuracy, useful in areas like emergency food assistance prediction^11^. Base learners can be homogeneous or heterogeneous.

Deep learning is a subset of machine learning that uses artificial neural networks to model and understand complex patterns in data^12^. It is a powerful and versatile approach that has revolutionized many fields, including computer vision, natural language processing, speech recognition^13^. As the field continues to evolve, deep learning holds great promise for addressing some of the most challenging problems in artificial intelligence and data analysis^14^. A hybrid model is a combination of two or more different models to leverage the strengths of each model^15^. A hybrid model is more accurate and robust than a single model, especially when dealing with complex or noisy data. Additionally, a hybrid model can provide more flexibility and adaptability, as it can be tailored to specific requirements by incorporating different techniques^15^. In this study, Explainable AI (XAI) is not merely applied as a technical visualization tool but as a critical component of scientific inquiry into model behavior. We investigate how post-hoc explainability methods, such as LIME, can systematically decompose the decision logic of complex hybrid models. This inquiry is essential for assessing whether the model’s predictive pathways align with established clinical knowledge or if they rely on spurious correlations, thereby determining the scientific validity and ‘right-for-the-right-reason’ nature of the predictions^16^. This is an important aspect of AI systems as it helps users understand and trust the AI models for deploying, especially in critical domains^16^, such as stunting prediction. XAI is important to reliance on intelligent machines^17^. The main goal of XAI is to enable users to understand the model’s behavior, appropriately trust the model’s predictions, and produce more explainable models^17^. Our study improves childhood stunting prediction using a novel and robust hybrid model with LIME for transparent, interpretable predictions of stunting severity. This fosters trust in multi-class classification (Normal, Moderate, and Severe) for nuanced WHO-based assessment and uses rigorous hybrid feature selection to enhance efficiency and performance. By integrating post-hoc explainability methods (LIME), this research moves beyond mere classification. It identifies the key risk factors, such as maternal education, household wealth, and access to clean water, that are pivotal for designing evidence-based, Sustainable Development Goals (SDG)-aligned interventions. Ultimately, this work provides a scalable framework for using artificial intelligence to safeguard the developmental potential of the next generation, ensuring that no child is left behind in the pursuit of health and nutritional equity. To this end, the current research tries to investigate and answer the following research questions.

1. What are the high-performing algorithms for stunting prediction?
2. To what extent does the proposed predictive model accurately identify the stunting?
3. What are the key determinant factors that contribute to stunting among children under five in Ethiopia?

## Related works

In this study, we analyze and evaluate various literatures from journals to attain knowledge about the problem. Also, we conduct a comprehensive literature review to gather existing knowledge on the factors influencing stunting.

Ayele et al.^1^ This study demonstrates the effectiveness of machine learning in accurately predicting childhood stunting in Ethiopia using the EDHS dataset from 2011 to 2016. The study used Random Forest, AdaBoost, XGBoost, and CatBoost, evaluating performance via accuracy, precision, recall, F1-score, and ROC-AUC. Random Forest achieved 97.746% accuracy. This study overcame a key limitation in previous stunting prediction models by developing a multi-class classification model that predicts stunting severity (severe, moderate, normal). The top risk factors contributing to stunting included the child’s age, maternal education level, birth order, household wealth index, mother’s BMI, breastfeeding duration, and access to clean water and sanitation. The findings provide critical insights for healthcare professionals and policymakers to implement targeted intervention strategies, ultimately reducing childhood stunting prevalence. These models often operate as ‘black boxes’, offering high predictive accuracy but limited interpretability, which hinders their utility for designing targeted public health policies. Our study moved beyond single ensemble models by implementing a hybrid (Extra Tree + MLP) model to achieve high predictive power across key multi-class categories, resulting in trustworthy and explainable models. The use of the 2019 EDHS data provides the most current risk factor assessment, ensuring the findings are immediately relevant to Ethiopia’s current public health strategy.

Wicaksono et al.^18^ Develops and evaluates an explainable machine learning framework to predict stunting among toddlers using simple anthropometric and demographic data, achieving an accuracy of 97.57% with XGBoost and providing actionable insights for early identification of at-risk children. XGBoost (97.57% accuracy) best predicted stunting, closely followed by Random Forest (97.28%) and Decision Tree (96.62%). SHAP analysis identified height as the most influential factor, then age, gender, and weight. Explainable machine learning, particularly XGBoost with SHAP, accurately and transparently predicts stunting using basic data, bridging the gap between modeling and public health insights. A limitation of this paper is its failure to specify the severity of stunting (severe or moderate), which could complicate clinical and public health interventions for affected children. We improved predictive power across key multi-class categories by using a hybrid model instead of single ensemble models.

Ntawuyirushintege et al.^3^ The study analyzed the prevalence of stunting among children under two years old in Rwanda between 2020 and 2024, revealing a substantial decline in national prevalence, but with persistent disparities across districts and sectors. The study found that the prevalence of stunting among children under 2 years old in Rwanda varied significantly across different districts and sectors, with some areas achieving significant reductions in stunting prevalence while others saw minimal change. Factors contributing to the decline include improved maternal health, socioeconomic conditions, and nutrition programs. Challenges remain, including infectious diseases and socio-cultural factors. Gahanga sector (Kigali) shows relatively high stunting (21.2% in 2024). The results of the study show trends in the prevalence of stunting among children under two years old in different provinces of Rwanda between 2020 and 2024. The study has several limitations, including the unavailability of data for 2021 due to COVID-19, a limited number of variables, and the lack of longitudinal follow-up of children.

Sisay et al.^4^ examined the prevalence and factors associated with childhood stunting in Ethiopia, using a large sample size of Ethiopian children, and found that 35.7% of children under five years old had stunting, with significant associations with the child’s sex, maternal age, community maternal education, household wealth status, and region. The key findings of the study were that the sex of the child, maternal age, and community maternal education were significantly associated with lower odds of stunting severity levels, whereas child age in months, household wealth status, maternal education, and region were significantly associated with higher odds of stunting severity levels. Similar to the work of Sisay et al., our study relies on cross-sectional data from the 2019 EDHS. Consequently, the relationships identified by our model are correlational rather than causal. This limitation is consistently considered when discussing the potential for predictive insights to inform public health strategies. Our study extended beyond single ensemble models by implementing a hybrid architectural model, which achieved high predictive power across key multi-class categories with trustworthy, explainable models.

In our study, this hybrid XAI model combining Extra Tree and MLP achieves 94% accuracy and robustness By critically evaluating the consistency of the LIME method, this study enhances the trustworthiness of the hybrid model. The model predicts multi-class stunting severity (normal, moderate, severe) for targeted interventions. The use of the 2019 EDHS data provides the most current risk factor assessment, ensuring the findings are immediately relevant to Ethiopia’s current public health strategy.

## Methods

### Data source and preparation

**Fig. 1:**
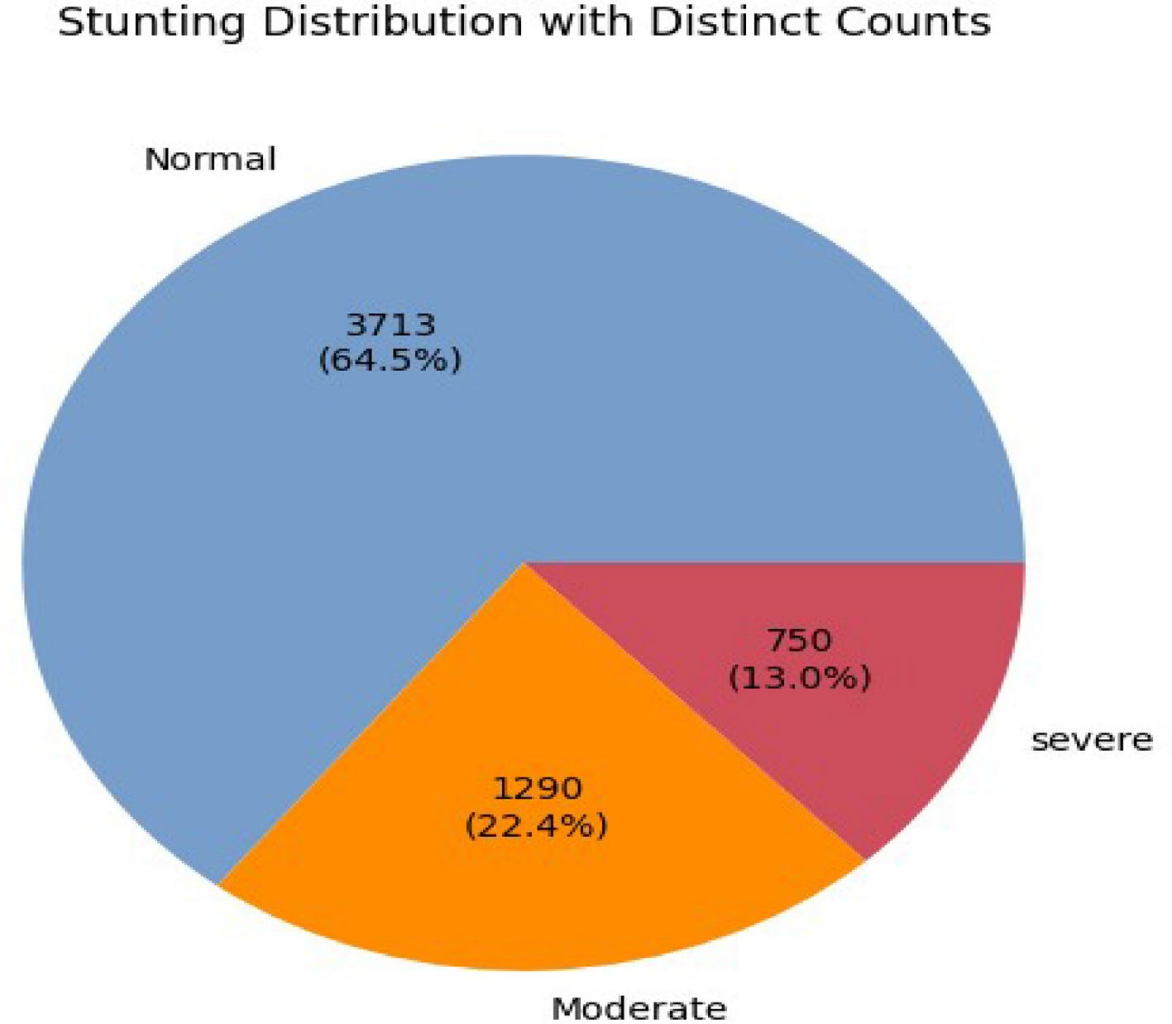
Stunting distribution, the original data from the Demographic and Health Surveys (DHS) Program website, before preprocessing.

The data for this study were obtained from the 2019 Ethiopian Demographic and Health Survey (EDHS), which is available on the Demographic and Health Surveys (DHS) Program website (https://dhsprogram.com/data/). While not publicly available, the data, which include demographic, socioeconomic, and health indicators, can be accessed upon registration, authorization, and reasonable request from the DHS Program. We focused on responses related to children under the age of five (Fig. 1). After cleaning the data by addressing missing values and removing duplicates, the dataset comprised 5753 instances with 21 features. The target variable, “stunting status,” was classified according to WHO standards into three categories: severely stunted (height-for-age Z-score < -3SD), moderately stunted (-3SD ≤ height-for-age Z-score < -2SD), and normal (height-for-age Z-score ≥ -2SD).

Children exhibited a 35.4% stunting prevalence, with moderate stunting affecting 22.4% and severe stunting affecting 13.0%. This highlights the urgent need for targeted interventions to combat malnutrition and foster healthy growth in children under five.

### Data preprocessing

Data pre-processing, which formats and organizes data for analysis, is crucial for machine learning algorithm performance. It eliminates noise and irrelevant features, streamlining model development^19^. If irrelevant, redundant, noisy, or unreliable data is present in the dataset and used for model development, achieving the desired outcome during the training phase becomes more challenging^20^. Data preprocessing involves cleaning, transforming, balancing, and selecting features to ensure that the data is structured and reliable for analysis and modeling. These steps are strategically chosen to create a robust predictive model from Demographic and Health Surveys (DHS) data for stunting in children under five.

### Data cleaning

Data cleaning is the process of filling in missing values and removing duplicate entries in a dataset. The goal is to ensure that the dataset is accurate, complete, and ready for analysis^19^. In this study, the dataset utilized in the model contained no missing values. The researchers handled missing data through an imputation method in STATA software (Version 17, StataCorp, https://www.stata.com). Checking the data for quality shows that there is redundancy of instances observed, which requires removal, as shown below. The original dataset contained 5753 instances, with 3713, 1290, and 750 classified as normal, moderate, and severe, respectively. 7 duplicate instances (6 normal, 1 moderate) were removed. For our study, we used text cleaning to remove noise and standardize input.

### Data transformation

Data transformation involves converting raw data into a format that is suitable for analysis and modeling^21^. Machine learning algorithms perform optimally with numerical input. Therefore, categorical data must be encoded into numerical representations. In this study, we employed dictionary mapping techniques for categorical feature encoding.

### Data balancing

The imbalanced class distribution problem in collected data can be addressed by adding or removing samples from the dataset through sampling techniques like Random Under-Sampling, Random Over-Sampling, SMOTE (Synthetic Minority Over-sampling Technique), ADASYN (Adaptive Synthetic Sampling), and Ensemble Techniques^22^. SMOTE works by creating synthetic samples of the minority class by interpolating between existing samples. This helps to increase the number of minority class samples and balance the dataset. The distribution of the class label was imbalanced. To get the best-performing model, the data must be balanced. To conduct this study, we used the synthetic minority over-sampling technique (SMOTE) to handle the class imbalance levels of the. SMOTE is effective in addressing imbalanced class distribution by generates synthetic data points by identifying nearest neighbors and creating new data points in between them. SMOTE is preferred because it helps avoid the loss of valuable information while balancing the class levels of the dataset and create diversity in the dataset^23^.

**Fig. 2:**
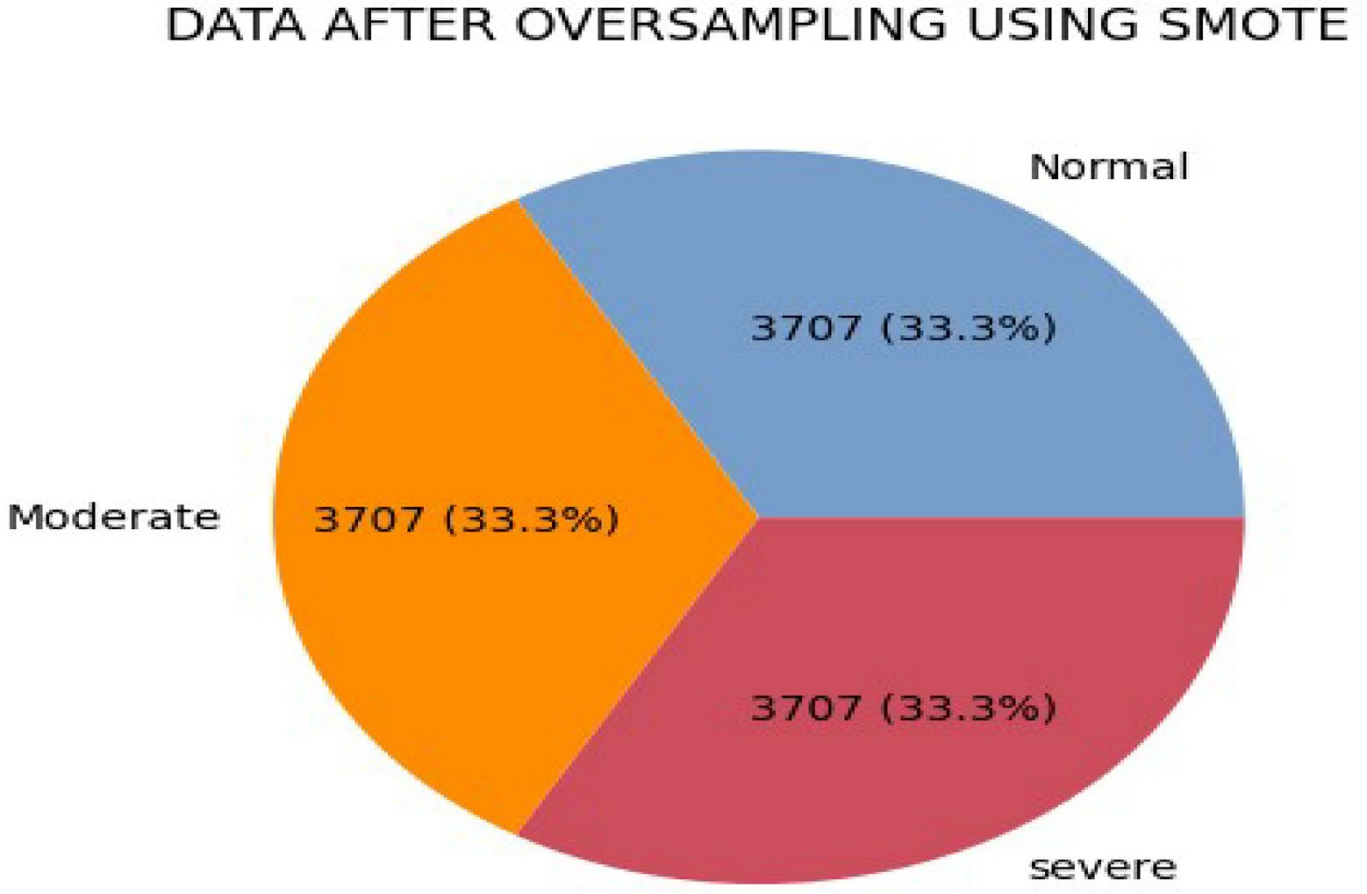
Data after oversampling, the dataset used for model development, after we applied preprocessing.

### Feature selection

Feature selection is a crucial step in machine learning and data analysis, where the goal is to identify and choose the most relevant features that contribute to the performance of a model^24^. By selecting the right features, we can improve the accuracy of our predictions, reduce over-fitting, and enhance the interpretability of the model^25^. There are various techniques available for feature selection, such as filter methods, wrapper methods, and embedded methods, each with its own strengths and weaknesses^26^.

Filter method feature selection in machine learning and data analysis helps in identifying the most relevant features^27^. This method involves ranking the features based on certain criteria, such as correlation with the target variable or statistical significance. By selecting only the most informative features, the model can be simplified and made more interpretable, while also potentially improving its performance. In filter method, mutual information, and chi-square test were done^28^. Mutual information measures information between two variables symmetrically and non-negatively. Wrapper-based feature selection is a popular method used in machine learning to determine the most relevant features for a given model^29^. Step forward and step backward are two common approaches within wrapper-based feature selection. Step forward selection starts with no features and adds them iteratively, evaluating model performance at each step. Step backward selection begins with all features and removes them iteratively, again evaluating performance. Wrapper methods leveraging the model’s predictive power to evaluate feature subsets. However, it is important to use wrapper methods judiciously and in conjunction with other feature selection techniques to ensure robust and generalizable model performance. Hybrid feature selection is a method that combines multiple feature selection techniques or metrics to identify the most relevant features for a given task^30^. It aims to overcome the limitations of individual feature selection methods and improve the overall performance and stability of the feature selection process. For this study, we used a hybrid feature selection method that combines filter and wrapper techniques to enhance the accuracy and efficiency of the model. This approach allowed us to identify the most relevant features while minimizing redundancy, ultimately improving predictive performance. We identified 17 critical features using hybrid feature selection for classifying the status of stunting. These features, culled from a comprehensive dataset, offer a nuanced understanding of the multifaceted factors contributing to stunting.

### Dataset split

The non-overlapping nature of the training and testing data in machine learning is essential for accurate model evaluation, preventing over-fitting, assessing generalization capabilities, and optimizing hyper-parameters^31^. In this study, to estimate the model’s performance on unseen data, we need to have a separate dataset. This is achieved by splitting our available datasets into training and testing. To decide the dataset split ratio for machine learning, there is no one-size-fits-all answer. The optimal split ratio depends on various factors, such as the size of the dataset, the complexity of the model, and the nature of the problem^31^. For this study, we used an 80/20 train test split. We then evaluate the model’s performance on the test set using metrics such as accuracy, precision, recall, and F1-score. These metrics provide a comprehensive understanding of the model’s ability to correctly classify instances.

### Select a predictive model

Following data preprocessing and splitting, the predictive model was built using ensemble and deep learning algorithms, specifically Random Forest, XGBoost, Extra Trees, MLP, and a Hybrid model. These algorithms were chosen for their proven effectiveness, adaptability to diverse data types, and ability to generate accurate predictions, particularly in capturing complex relationships within tabular datasets.

Random Forest: As noted in ^32^, Random Forest trees are constructed independently using a random subset of features. They can handle both categorical and continuous data, making them suitable for tabular datasets. Random Forest models are robust against over-fitting and demonstrate good generalization to unseen data, particularly in high-dimensional datasets^33^.

Extreme Gradient Boosting (XGBoost): As pointed out by^34^, XGBoost is a powerful algorithm that can handle both categorical and numerical data, making it suitable for tabular datasets. XGBoost employs regularization techniques to enhance model generalization and prevent over-fitting. It can handle high- dimensional datasets efficiently by using gradient boosting, which combines weak learners to create a strong learner. XGBoost has built-in regularization techniques to prevent over-fitting, making it suitable for high-dimensional datasets. XGBoost provides a feature importance analysis, which helps identify the most relevant features in the dataset.

Extra Tree: As noted in ^35^, The random feature selection helps to reduce over-fitting by introducing randomness and diversity among the decision trees. It can handle high-dimensional datasets efficiently by randomly selecting feature splits, reducing the impact of irrelevant or noisy features. Extra Tree is less prone to over-fitting compared to traditional decision trees, making it suitable for high-dimensional datasets. Extra Tree is suitable for tabular datasets as it can handle both categorical and numerical data.

Multilayer Perceptron (MLP): pointed out by^36^ MLP is suitable for classification tasks. It is a type of feed forward artificial neural network that can be used to classify input data into different categories. The MLP consists of multiple layers of nodes, each connected to the next layer, and uses non-linear activation functions to process the input data. With proper training and optimization, the MLP model can effectively classify data into distinct classes, making it a powerful tool for various classification tasks.

Hybrid model: A hybrid model is more important to increase the performance of a model than an individual model^37^. In our study, to optimize performance, we have developed a hybrid model that leverages the strengths of two top-performing models, MLP and Extra Tree, aiming to create a more robust and accurate predictive model. Through this hybrid approach, we can harness the strengths of each model to mitigate its weaknesses, resulting in a more reliable predictive model. By combining the MLP and extra tree models, we can leverage their complementary strengths to enhance the performance of the model.

### How does the hybrid model work explained as follows

To construct the hybrid architecture, we combined an Extra Trees Sequential Ensemble Method with an MLP model. First, we define ExtraTreesClassifier and MLPClassifier models from scikit-learn with appropriate parameters. Next, we directly utilize the ExtraTreesClassifier for feature importance or predictions as a meaningful transformation process, avoiding a FunctionTransformer. If feature augmentation is desired, we extract the Extra Trees model outputs to enhance the feature set before input to the MLP model. Subsequently, we construct a pipeline to sequentially combine the models. This pipeline includes the Extra Trees classifier processing input data, potentially yielding new feature representations for the MLP classifier. After defining the pipeline, we train the hybrid model using the fit method and training data. Once trained, we generate predictions on the test set using the pipeline’s predict() method. Finally, we evaluate performance by generating and displaying a classification report using the classification_report function, which provides precision, recall, F1 score, and support for each class. This approach ensures a structured, reproducible, and clear implementation of the hybrid model.

### Parameter tuning

Parameter tuning in machine learning refers to the process of selecting the optimal values for the hyper-parameters of a model^38^. Hyper-parameters are parameters that are not learned from the data, but rather set by the user before training the model. The goal of parameter tuning is to find the combination of hyper-parameter values that result in the best performance of the model on a given task^39^. Adjusting the hyper parameters can improve the performance of the model and ultimately achieve better results, techniques for hyper parameter tuning, including grid search, random search, and Bayesian optimization^40^. Grid Search exhaustively searches all possible combinations of hyper parameters within the defined range. Random Search randomly samples hyper parameter combinations from the defined range. Bayesian Optimization uses probabilistic models to guide the search process and focus on promising regions of the hyper parameter space. In this study, we used a grid search parameter tuning method to optimize model performance of the model. Grid Search is the classic way for hyper-parameter tuning. Grid search finds the best solution but is slow due to many combinations, needing high computational power, making it expensive. We used L2 weight decay on dense layers and Dropout in every hidden block for the better performance of the MLP model. To achieve this, we incorporate the dense layers, setting it to L2 regularization with a weight decay factor. This encourages smaller weights, preventing overfitting and improving generalization. Furthermore, we introduce Dropout layers after each dense layer in the hidden blocks, with a dropout rate. This randomly deactivates neurons during training, forcing the network to learn more robust features and reducing co-adaptation between neurons. The combination of L2 weight decay and Dropout helps to regularize the MLP model, leading to enhanced performance on unseen data and mitigating the risk of overfitting. The following Table illustrates the selected model with the corresponding hyper parameter (Table 1).

**Table 1:** Hyper parameter grid for the selected model, used as a setting to train the model.

No	Algorithm	Hyper Parameter name	Hyper parameter grid	Best parameter
1	Random forest	n_estimators	[ 100, 500, 1000]	500
		Criterion	[‘gini’, ‘entropy’]	Entropy
		max_depth	[None, 10]	None
		min_samples_split	[5, 10]	5
		min_samples_leaf	[2, 5]	2
		max_features	[‘auto’, ‘sqrt’]	Auto
2	Extreme gradient boosting	n_estimators	[ 100, 500, 1000]	100
		learning_rate	[0.1,0.3, 0.5,0.7, 1.0]	0.5
		max_depth	[None, 10]	None
		Subsample	[0.9, 0.8, 0.7]	0.9
		Gamma	[0, 0.1, 0.5]	0.1
		min_chlid_weight	[1, 3, 5]	3
3	Extra tree	n_estimators	[ 100, 500, 1000]	1000
		Criterion	[‘gini’, ‘entropy’]	Gini
		min_samples_split	[5, 10]	5
		min_samples_leaf	[2, 5]	2
		max_depth	[None, 10]	None
		max_features	[‘auto’, ‘sqrt’]	Auto
4	MLP	Activation Function	[Softmax, relu]	Softmax
		Learning Rate	[0.001, 0.0001, 0.00001]	0.001
		Number of layers	[3, 4, 5]	5
		hidden_layer_sizes	[50,100]	50
		Dropout rate	[0.2, 0.3, 0.4]	0.3
		l2_regs	[0.001, 0.0001]	0.001
		num_dense_layers	[1, 2]	2
		Batch size	[8, 16, 32]	8

### Model evaluation

Model evaluation is a crucial step in the machine learning process, which allows to assess of the performance and effectiveness of a model^41^. The most common objective-based evaluation metrics used include accuracy, precision, recall, cross-validation, confusion matrix, and F1-score. A confusion matrix is a vital tool for evaluating the classification performance of a model by summarizing predicted and actual values^37^. Analyzing the matrix allows for the calculation of accuracy, precision, recall, and F1 score, which are crucial metrics for model evaluation^42^. Regular review and interpretation of the matrix helps identify model weaknesses for necessary improvements. The matrix displays actual and predicted classes in rows and columns, with diagonal elements showing correct predictions. Off-diagonal elements reveal frequently misclassified classes, highlighting areas for model enhancement. The confusion matrix allows for analysis of the model’s performance for each class^43^.

- Identify classes with high misclassification rates (high values off the diagonal).
- See how often the model confuses specific classes (e.g., Class 1 and Class 2).

Analyzing these errors can improve model performance by tuning the classification algorithm or collecting more data to better differentiate classes (Table 2).

**Table 2:** Is an example of a confusion matrix for a study that has three classes.

	Predicted class			
		Class 1	Class 2	Class 3
Actual Class	Class 1	C₁₁	C₁₂	C₁₃
	Class 2	C₂₁	C₂₂	C₂₃
	Class 3	C₃₁	C₃₂	C₃₃

- C11, C22, and C33 represent the number of correctly classified instances for each class.
- Others represent the number of instances that were misclassified between the respective classes.

As per the learning result correctly or incorrectly classified instances are further grouped as the following^42^.

- **True positives (TP)** - True positives occur when both the actual and predicted classes are positive. For example, C11, C22, and C33 are true positives for their respective classes specifically, C11 is a true positive for Class 1.
- **False positives (FP)** - False positives occur when the actual class is negative, but the predicted class is positive, or the sum of values of the corresponding column, except for the TP value. E.g., for Class 1, false positives are C21 and C31.
- **False negatives (FN)**- False negatives happen when the actual class is positive, but the predicted class is negative, which means the sum of values of the associated rows, except for the TP value. E.g., for Class 1, C12 and C13 are false negatives.
- **True negatives (TN)**- True negatives are cases when the actual class is negative, and the predicted class is also negative it means the total of all columns and rows, excluding the values of the class for TP, FP, and FN. E.g. for Class 1 C22,C23,C32, and C33 is TN.

1 $${\mathrm{Accuracy}}={\mathrm{~}}\frac{{\left( {{\mathrm{TP~}}+{\mathrm{~TN}}} \right)}}{{\left( {{\mathrm{TP}}+{\mathrm{TN}}+{\mathrm{FP}}+{\mathrm{FN}}} \right)}}$$

2 $${\mathrm{Precision~}}={\mathrm{~}}\frac{{{\mathrm{TP}}}}{{\left( {{\mathrm{TP~}}+{\mathrm{~FP}}} \right)}}$$

3 $${\mathrm{Recall}}=\frac{{{\mathrm{TP}}}}{{\left( {{\mathrm{TP~}}+{\mathrm{~FN}}} \right)}}$$

4 $${\mathrm{F}}1 - {\mathrm{score}}=\frac{{\left( {2{\mathrm{~*~Precision~*~Recall}}} \right)}}{{\left( {{\mathrm{Precision~}}+{\mathrm{~Recall}}} \right)}}$$

### Experiment

**Fig. 3:**
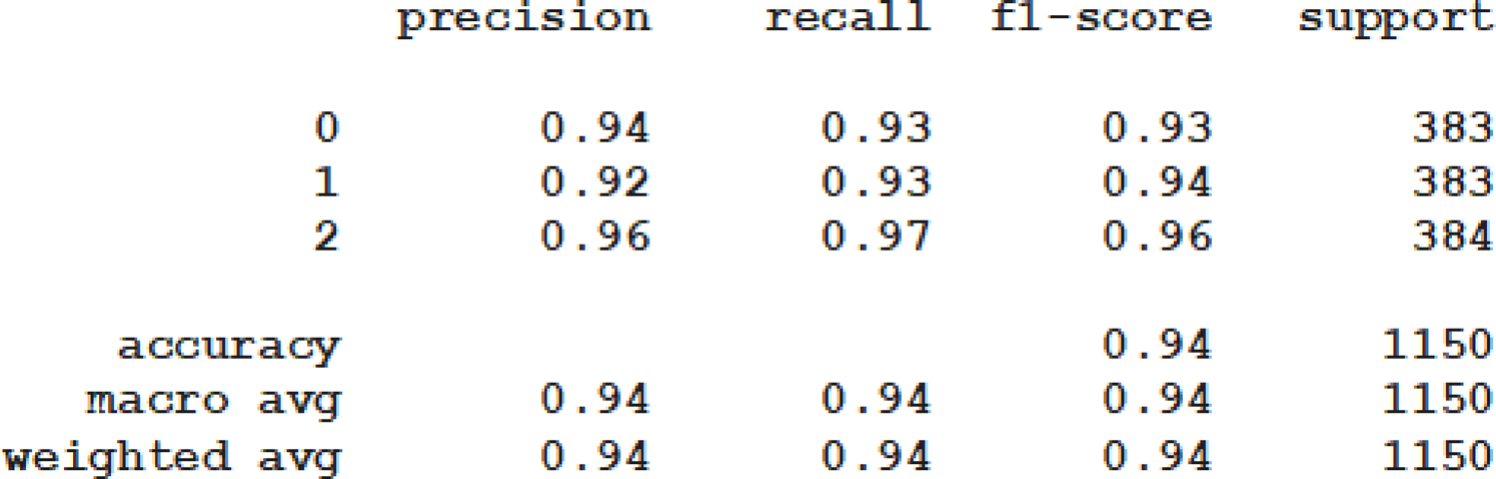
Hybrid model performance: a predictive performance of the model by class and average.

Hybrid feature selection identified 17 critical features for classifying stunting status. We evaluated prediction performance using five experiments: Random Forest, Extra Tree, MLP, Extreme Gradient Boosting, and a hybrid model. Model training required a dataset of 11,121 instances categorized as normal, moderate, or severe stunting. Predictive model construction involved three phases: training and testing^15^. During training, data is repeatedly shown to the model to elicit the desired response. In the testing phase, the trained model is applied to unseen data to evaluate its performance^44^. Therefore, the predictive model should be developed by partitioning the complete dataset into training and testing subsets. As a result, out of the total 11,121 datasets, 80% and 20% were designated for training and testing, respectively. The hybrid model predicts stunting with an average of 94% across accuracy, precision, recall, and F1-score. The ROC curve and cross-validation performance is 98%, demonstrating strong predictive power and generalization. The model had optimal performance with k = 5 for cross-validation. ROC curve and cross-validation evaluate the effectiveness of class distinction and model robustness (Fig. 3).

**Fig. 4:**
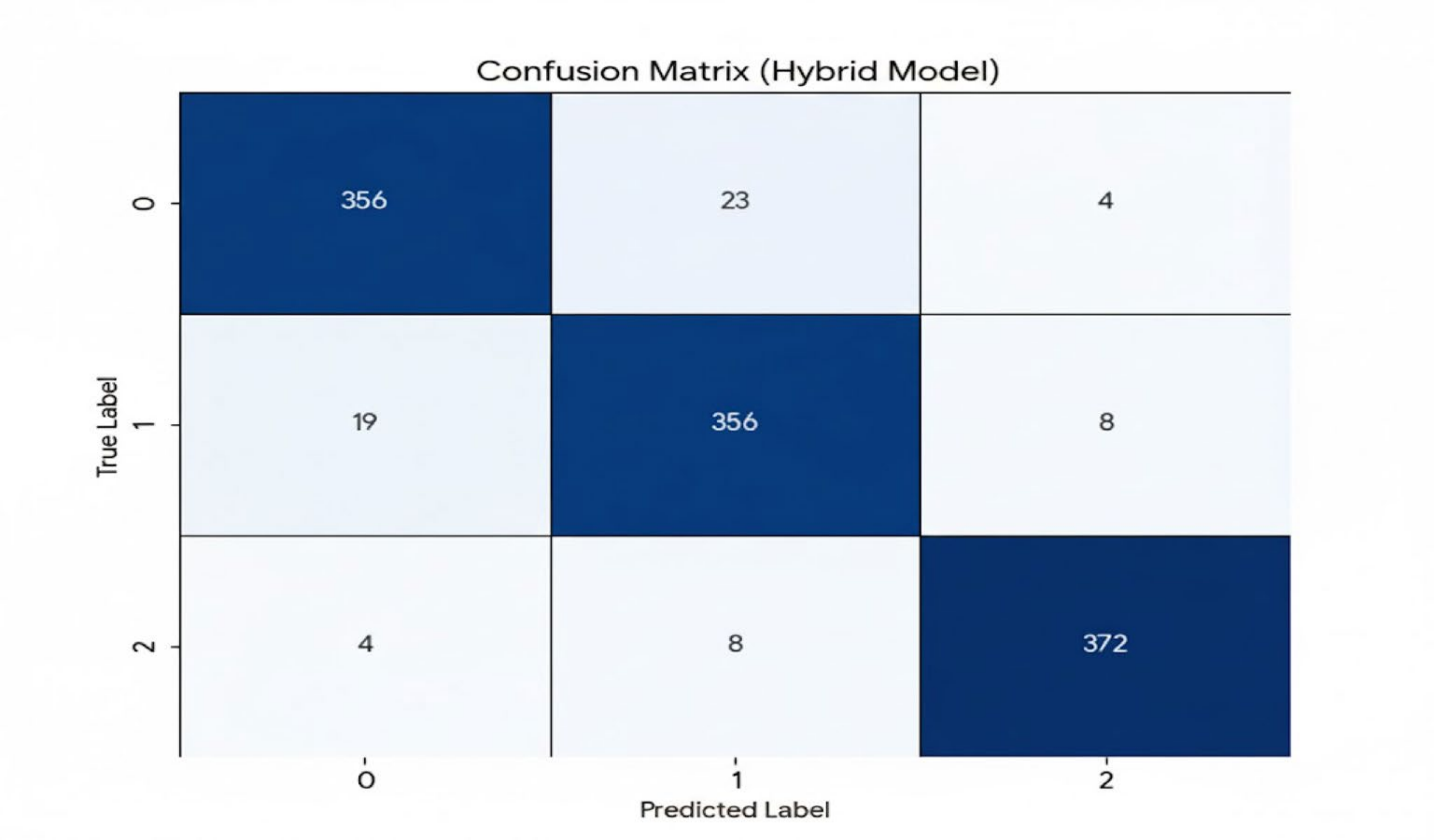
the hybrid model’s confusion model visualizes classification accuracy using true and predicted labels.

The confusion matrix evaluates a multi-class classification model with three classes (0, 1, and 2). The model demonstrates strong performance, indicated by a high number of correct predictions along the diagonal. The matrix below shows the classification results of the hybrid model analysis based on a test set of 1150 samples (Fig. 4).

- Class 0 (Normal): 383 total instances, with 356, 23, and 4 classified as a correct class, misclassified as Moderate, and misclassified as severe respectively.
- Class 1 (Moderate): 383 total instances, with 356, 19, and 8 classified as a correct class, misclassified as Normal, and misclassified as severe respectively.
- Class 2 (severe): 384 total instances, with 372, 4, and 8 classified as a correct class, misclassified as Normal, and misclassified as Moderate respectively.

A granular analysis of the hybrid model’s confusion matrix reveals systematic misclassification patterns, particularly at the boundaries of stunting severity. Specifically, while the model correctly identified 356 instances of moderate stunting, 19 cases were misclassified as Normal and 8 as Severe. Conversely, for the severe category, 8 instances were misidentified as Moderate. These boundary errors are clinically significant. The model’s occasional confusion between these adjacent categories suggests a degree of predictive uncertainty in cases with borderline anthropometric measurements, which could be attributed to the inherent noise in survey-based data collection.

**Fig. 5:**
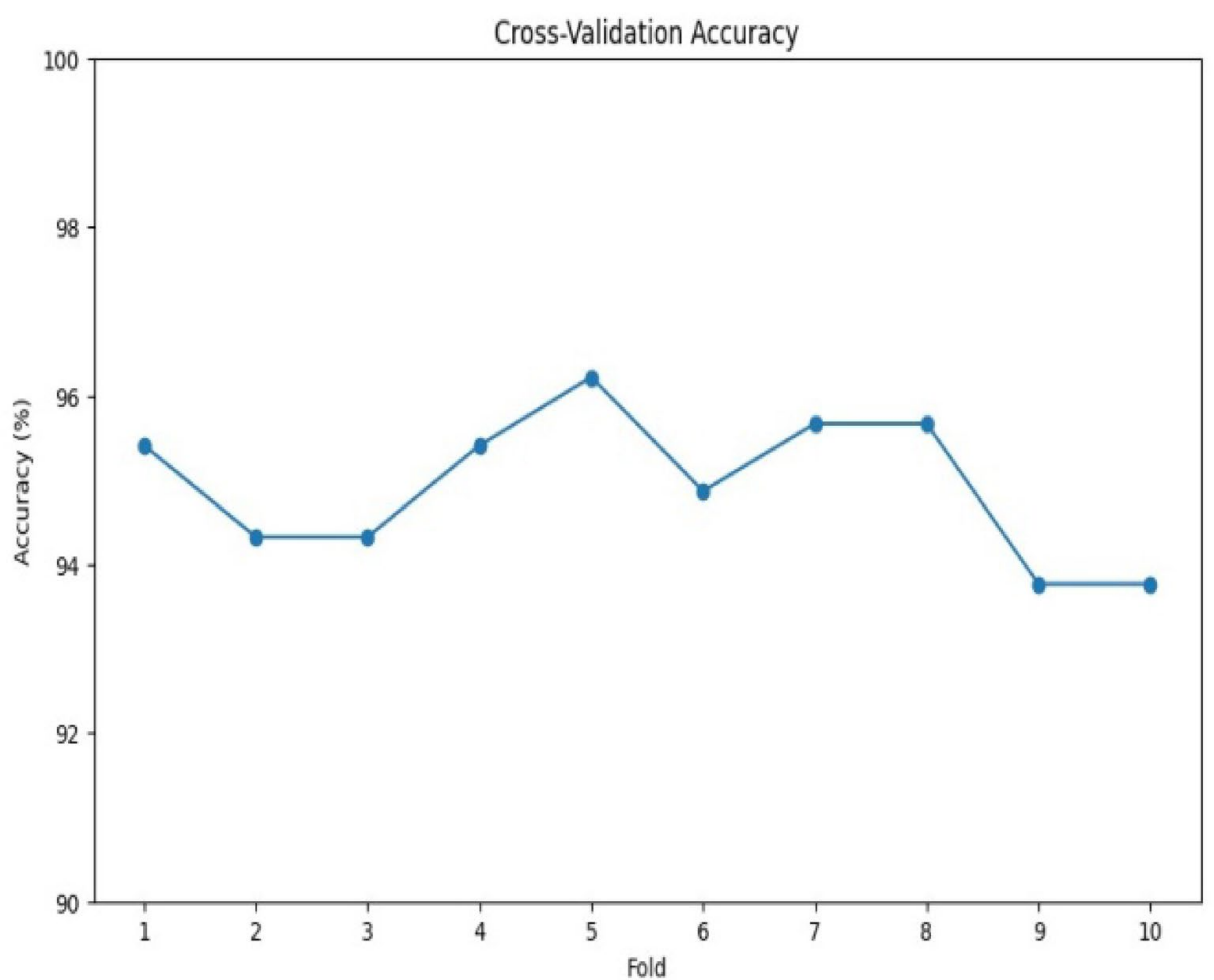
the hybrid model cross-validation performance, assess the performance and generalization ability of a hybrid model.

Cross-validation is a performance evaluation metric that is used to assess the performance and generalization ability of a model. It involves dividing the dataset into multiple subsets (k-fold)^45^. K-fold cross-validation divides the dataset into k equal folds. The model undergoes k training and evaluation cycles, with each fold serving as the validation set once, using the remaining k-1 folds for training. Averaging the performance metrics from each cycle provides an overall performance estimate. Cross-validation offers a more reliable performance assessment, aids in over-fitting detection, and facilitates hyper-parameter tuning. As shown in Fig. 5, the hybrid model’s k-fold cross-validation performance is strong, exhibiting high accuracy and good stability across data splits, indicating robust generalizability. While the model appears robust, analyzing lower-performing folds could reveal optimization opportunities. Optimal performance was achieved with k = 5.

**Fig. 6:**
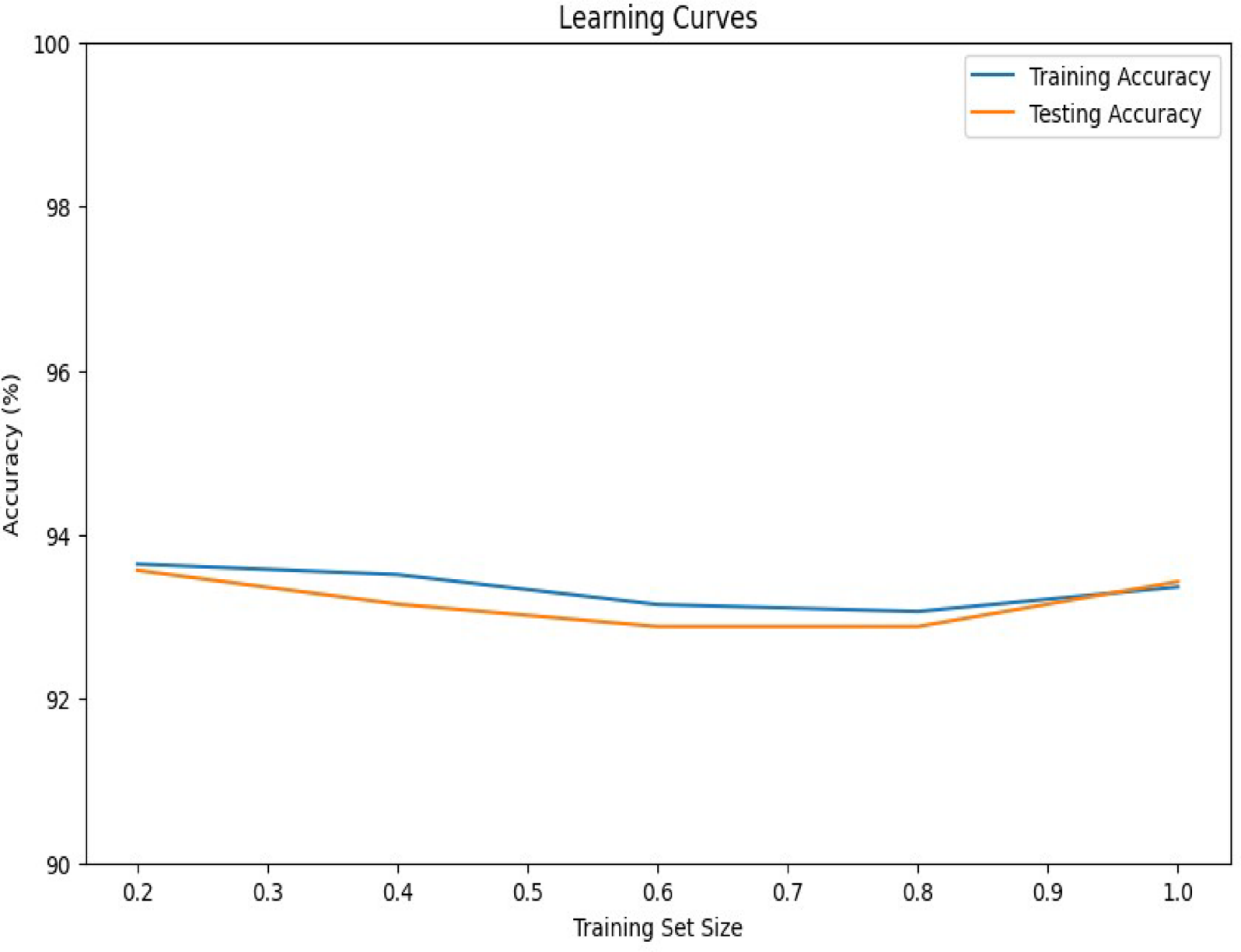
Learning curve for hybrid model, is a graphical representation that serves as a vital diagnostic tool to evaluate a model’s performance and learning behavior.

The graph shows consistently high training (blue) and testing (orange) accuracies, both exceeding 94% across varying training set sizes, indicating strong performance of the hybrid model for this classification task and low bias. The close proximity of the training and testing accuracy curves suggests good generalization and minimal over-fitting (low variance). The curves plateau rapidly, showing diminishing returns in accuracy with increasing training data. A slight increase in testing accuracy with the full training set suggests a marginal benefit to utilizing all available data, achieving peak testing accuracy. The following figure depicts the learning curves for the hybrid model (Fig. 6).

### Model comparison

Model comparison in machine learning is a critical step in the development and evaluation of predictive models^16^. By comparing the performance of different models, the researcher can determine which algorithm is best suited for a particular problem. This process involves training multiple models on the same dataset and evaluating their performance using metrics such as accuracy, precision, recall, and F1 score^46^. Additionally, techniques such as cross-validation and hyper-parameter tuning can be used to further refine and compare the models. Ultimately, model comparison allows for the selection of the most effective algorithm for a given task, leading to improved predictive accuracy and performance. The researchers did six experiments to create a predictive model for stunting using various algorithms and compare its performance to select the optimal model (Table 3).

**Table 3:** The overall performance of an experiment using objective performance metrics.

Evaluation metrics	Extra tree (%)	Random forest (%)	XGBoosting (%)	MLP (%)	Hybrid (%)
Accuracy	92	90	90	92	**94**
Precision	92	90	90	92	**94**
Recall	92	90	90	92	**94**
F1_score	92	90	90	92	**95**
Cross-validation	95	93	92	95	**98**
ROC	95	94	94	96	**97**

### Hybrid model performance with statistical confidence

Confidence Intervals (CIs) play a crucial role in Machine Learning (ML) because they help quantify the uncertainty associated with model estimates, especially performance metrics calculated on limited test data^47^. The study assessed the effectiveness of the hybrid model using several performance metrics, which are now reported with the context of statistical reliability.

- **Average Performance Metrics**- The model maintained a consistent average of 94% across precision, recall, and F1-score. Reporting these with confidence intervals would further validate that the model’s high performance is not due to a specific random split of the data.
- **Cross-Validation Reliability**- The model achieved an optimal cross-validation performance of 98% using k = 5 folds. This high percentage demonstrates strong predictive power and the ability to generalize across different data subsets. The stability shown in the cross-validation graphs suggests that any calculated confidence interval would be narrow, indicating low variance and a robust model.

### Here is the data required to calculate the confidence intervals

- **Total Test Set Size (*n*)-** 1150 samples.
- **Performance Metric (*P*)**: 94% (or 0.94) accuracy for the hybrid model.
- **Confidence Level**: Typically set at 95%, which corresponds to a **z-score (*z*) of 1.96**.
- For classification accuracy, you can use the **Normal Approximation Interval** formula.

$${\mathrm{CI}}=P \pm z \times \sqrt {\frac{{P(1 - P)}}{n}}$$

- Calculate the Standard Error (SE):

$$SE=\sqrt {\frac{{0.94 \times (1 - 0.94)}}{{1,150}}} =\sqrt {\frac{{0.94 \times 0.06}}{{1,150}}} \approx 0.007$$

- Calculate the Margin of Error:

1.96 × 0.007 ≈ 0.0137 (or 1.37%).

Final CI: 94%±1.37%. This results in CI of **[92.63%**,** 95.37%]**.

### Risk factor analysis

In this study, to analyze risk factors, we used feature importance, which measures the contribution of each feature in predicting the target variable and can guide feature selection. Feature importance helps to identify the most influential features in a predictive model and understand their relative importance^15^. Employing a top-performing hybrid model, which consistently outperformed others across all evaluation metrics, we identified key risk factors for stunting using feature importance techniques. The feature with the highest importance value was considered the most determinant, and the feature with the lowest, the least. The figure below illustrates the hybrid model’s feature importance.

**Fig. 7:**
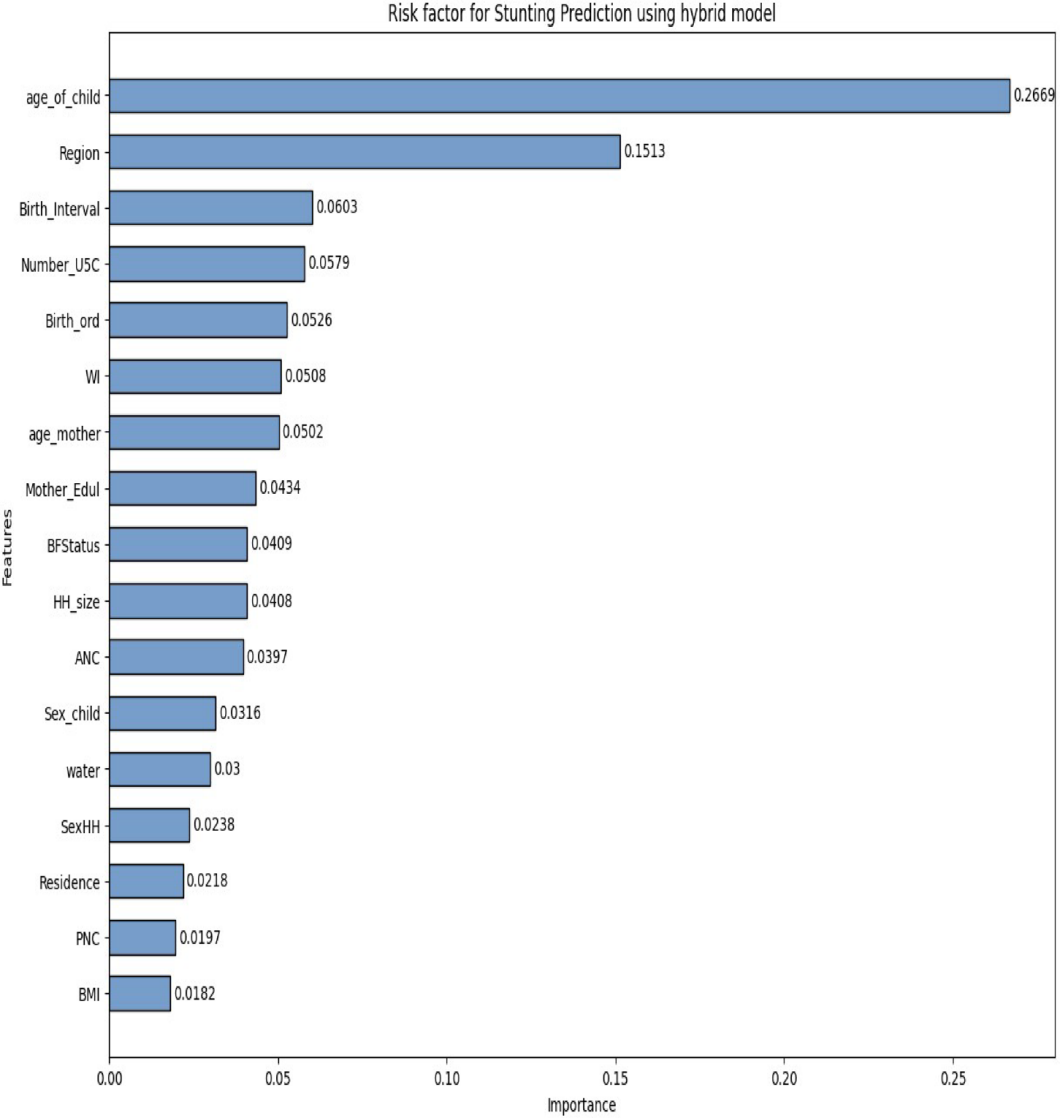
The hybrid model feature importance ranks features used in model development by their importance scores to analyze risk factors for child stunting.

Figure 7 illustrates the feature importance scores derived from a hybrid model used for predicting stunting risk factors in children. Each bar represents a specific feature’s contribution to the predictive capability of the model, with the height of the bar indicating the relative importance of that feature. The most significant predictor is age_of_child, with an importance score of 0.2699, suggesting that the age of a child plays a crucial role in determining stunting risk. Following this, Region and Birth_Interval also show notable importance scores of 0.1513 and 0.0603, respectively, indicating that geographical factors and spacing between births are relevant in assessing stunting risk. Other features, such as number of children under five in the household (Number_USC), Birth_ord, and Mother_Edu, contribute moderately to the model’s predictions. Features like Sex_child, Residence, and BMI appear to have lesser influences, as indicated by their lower importance scores. This distribution of feature importance highlights which factors are most critical for understanding and addressing stunting risk.

The analysis of feature importance in the stunting risk prediction model reveals critical insights into the factors influencing child growth outcomes. The prominence of age_of_child underscores the need for age-specific interventions and policies aimed at combating stunting. Additionally, the influence of regional factors and birth intervals suggests that targeted strategies should consider local contexts and family planning practices. By identifying and prioritizing these significant risk factors, stakeholders can better allocate resources and design effective interventions to prevent stunting. The model’s ability to quantify the importance of various features not only enhances understanding but also guides future research and public health initiatives aimed at improving child nutrition and health outcomes.

## Use of explainable artificial intelligence

During the study, XAI was developed with input from domain experts to improve model understanding and decision-making. To understand how the prediction works is crucial for making informed decisions and improving the model. XAI is used in stunting prediction for several reasons. Firstly, it provides transparency and interpretability in the decision-making process, which is crucial when consequences or risks are significant. By understanding how the AI model arrives at its predictions, stakeholders can have confidence in the results and make informed decisions about resource allocation and distribution. Secondly, XAI helps to identify biases and errors in the AI model, which is particularly important in the context of stunting. By leveraging XAI, stakeholders can make more informed and equitable decisions to effectively address stunting. Generally, XAI enhances the effectiveness of stunting prediction models by ensuring they are interpretable, trustworthy, and actionable, ultimately leading to better health outcomes for children.

### Interpretability methods to explain black-box model

There are various interpretability techniques like Local Interpretable Model-Agnostic Explanations (LIME), Explain Like I’m 5 (ELI5), and Shapley Additive Explanations (SHAP), which can be used with any black box model^17^. In this study, we used the LIME method, which is one of the most popular interpretability methods for black-box models^48^. Following a simple yet powerful approach, LIME can generate interpretations for single prediction scores produced by any classifier^17^. For any given instance and its corresponding prediction, simulated randomly-sampled data around the neighborhood of the input instance, for which the prediction was produced, are generated. Subsequently, while using the model in question, new predictions are made for generated instances and weighted by their proximity to the input instance^48^. Figure 5 illustrates the use of the LIME method to explain the prediction of stunting.

**Fig. 8:**
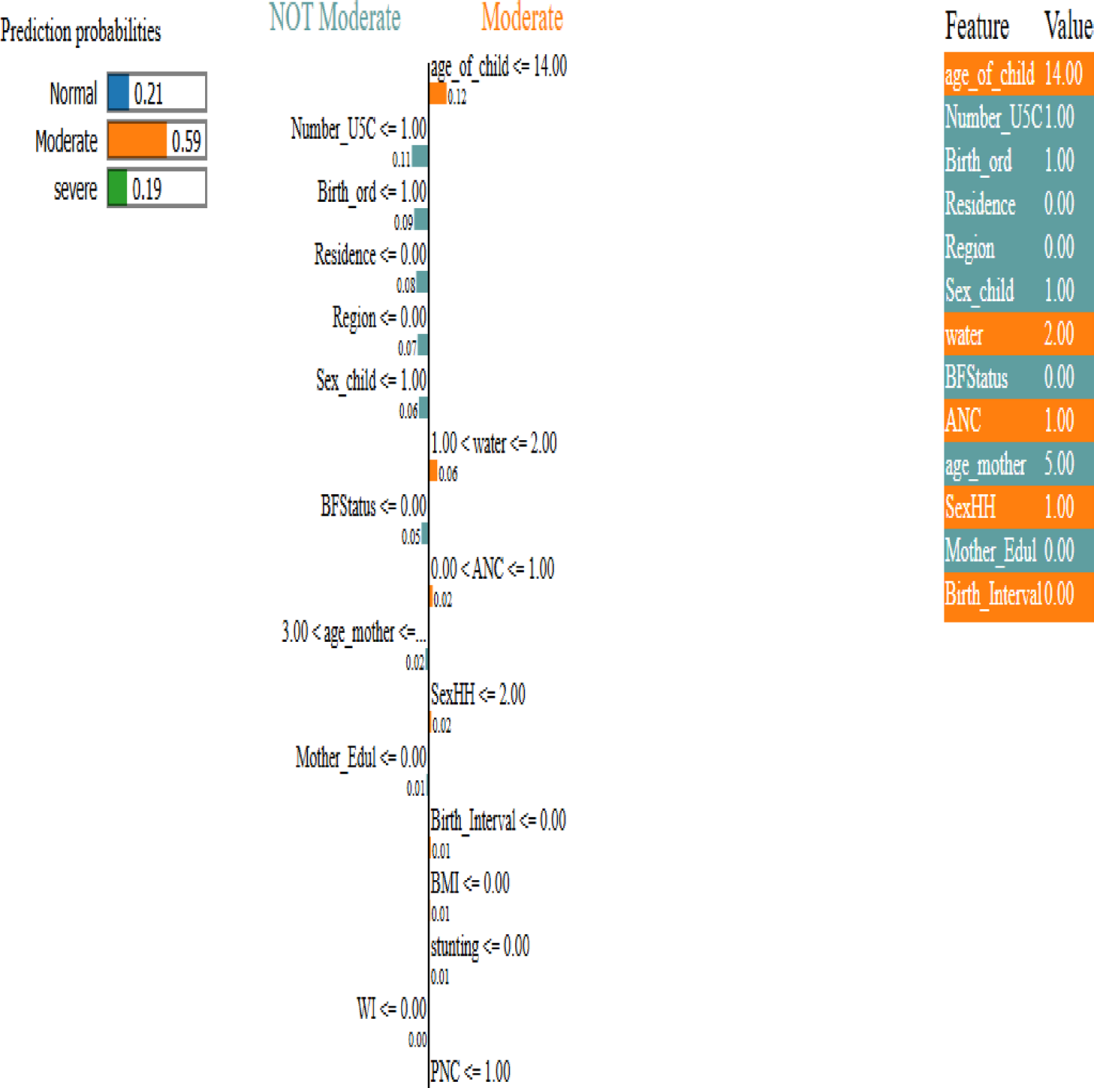
XAI explains hybrid model predictions, helping users understand the prediction process and the certainty of each classification.

In the visual representation displayed in Fig. 8 above, the left side illustrates the prediction probabilities for a specific instance across different classes. It shows probabilities of 21%, 59%, and 19% for the normal, moderate, and severe classes, respectively. This breakdown helps us understand the level of certainty associated with each classification. Highlighting the high probability in the moderate class enhances confidence in decision-making based on the predicted outcomes. The middle diagram illustrates the significance of each feature in contributing to the prediction of a particular class based on their respective weight. Features like age_of_child, water, ANC, and SexHH are highlighted as key factors that influence the classification of instances into the moderate class, XAI confirming the above risk factor analysis. Understanding the importance and impact of these specific features is crucial in accurately predicting and addressing vulnerabilities within the given dataset. By prioritizing these features in the analysis and decision-making process, we can better identify and mitigate potential risks associated with the identified class. On the right side of the diagram, each feature is represented along with its corresponding feature importance value, as well as the specific class to which it belongs, denoted by distinct color highlights. This visual representation allows for a clear and intuitive understanding of the relationships between the different features and their respective classes. By visually mapping out this information, stakeholders can easily identify patterns and trends within the data, leading to more informed decision-making processes. The use of color to differentiate between classes also enhances the overall clarity and readability of the diagram, making it a valuable tool for analysis and interpretation.

## Discussion

We constructed an explainable hybrid predictive model using 11,121 instances and 17 features. While we developed the model using a balanced dataset, we evaluated it on the original dataset to avoid inflating performance metrics. This study addresses three research questions, and we discuss our answers to these questions. The Feature Augmentation Hybrid model (Extra Trees + MLP) accurately predicts stunting (94% accuracy), offering a reliable tool for early screening and malnutrition prevention.

**RQ1:** “What are the high-performing algorithms for stunting prediction?” To answer this question, five experiments were conducted on the selected model, including random forest, extreme gradient boosting, MLP, extra tree, and a hybrid model. The experiments revealed that the hybrid demonstrates better performance.

**Complementary Strengths:** Ensemble methods like Extra Tree excel at capturing non-linear relationships and high-order feature interactions, offering strong performance by averaging the results of multiple decision trees. Deep learning models, specifically the Multilayer Perceptron (MLP), are adept at learning complex, hierarchical feature representations that may be missed by shallow models. The hybrid approach successfully leverages the robust, non-parametric modeling of the Extra Tree with the deep feature extraction capability of the MLP, resulting in a more generalized and robust model than either component could achieve alone.

**Robustness in Multi-Class Classification:** Existing literature often focuses on binary classification. The hybrid model’s success in handling multi-class classification (Normal, Moderate, and Severe) demonstrates its superior ability to distinguish the subtle boundaries between different severity levels, providing a more clinically relevant tool for public health officials.

**RQ2:** “To what extent does the proposed predictive model accurately identify the stunting? Our central inquiry was to evaluate the proposed predictive model’s ability to deliver accurate classification. To assess its predictive performance, a dedicated experiment was conducted. The results demonstrated that the hybrid model achieved an average accuracy of 94%, indicating a strong alignment between the system’s recommendations and actual conditions.

The hybrid model achieved a 94% average accuracy, meaning it correctly classified 94 out of 100 children into their actual stunting category (Normal, Moderate, or Severe). This high accuracy indicates a strong alignment between the model’s recommendations and actual conditions, making it suitable for public health applications. Specifically, the model can reliably identify at-risk children, prioritize severe cases for immediate intervention, and provide trustworthy predictions to policymakers and healthcare practitioners.

**RQ3:** “What are the key determinant factors that contribute to stunting among children under five in Ethiopia?” To answer this question, feature importance techniques were used on all the features that we used to develop the predictive model, and the potential attributes used to determine stunting were determined based on a hybrid model. As a result, age_of_child, Region, Birth_Interval, and Number_U5C are identified as the first, second, third, and fourth risk factors for stunting.

The findings of this study have direct and significant implications for improving public health policy and the effectiveness of nutritional programs in Ethiopia.

### Epidemiological factors and risk identification

- **Precision Policy**: The integration of Explainable AI (XAI) provides the crucial evidence base for policy decisions. The ability to locally explain why a child is predicted to be severely stunted allows policymakers to move beyond correlation to causation, ensuring that new policies are directly focused on mitigating the highest-impact factors. This shifts policy from reactive, national-level planning to proactive, evidence-based regional and local implementation.
- **Clinical Intervention**: Health clinics can use the model’s multi-class prediction (Normal, Moderate, and Severe) to prioritize resource allocation. Children predicted to be severely stunted can be immediately enrolled in therapeutic feeding programs, while those moderately stunted can be directed toward supplementary feeding and intensive parental counseling.
- **Public Health Intervention**: Programs must prioritize improving the quality and safety of complementary feeding starting at six months, including micronutrient supplementation and food fortification, particularly in high-risk regions.
- **Regional Resource Prioritization**: Policy should integrate the model’s high reliance on the Region to implement differential resource allocation. High-prevalence regions should receive increased funding for Water, Sanitation, and Hygiene (WASH) programs, as well as improved access to maternal and child healthcare, recognizing that stunting is a multi-sectoral issue rooted in geographic inequity.
- **Family Planning Integration**: The importance of Birth_Interval highlights the need to integrate nutritional programs with family planning services. Counseling on the health risks associated with short birth intervals must be a core component of both maternal and child health visits to prevent maternal depletion and subsequent stunting.

### Utility for public health decision-making

- **Proactive Screening**: Moving beyond retrospective prevalence tracking, this high-accuracy hybrid model (94% accuracy) allows for the proactive identification of children at risk of severe or moderate stunting before growth failure becomes irreversible.
- **Resource Allocation**: In resource-limited settings like Ethiopia, XAI (LIME) helps decision-makers understand why certain groups are at risk. This transparency ensures that interventions such as clean water initiatives or maternal education programs are directed toward the specific factors driving stunting in a particular sub-population.
- **Bridging the AI-Public Health Gap**: By providing explainable outputs that align with clinical knowledge, the model builds the necessary trust for healthcare workers to adopt AI-assisted tools in field assessments.

While the hybrid model achieves high overall accuracy (94%), its practical relevance for public health depends on understanding these classification nuances. The systematic misclassification between Moderate and Severe stunting highlights the model’s limitations in distinguishing between degrees of chronic undernutrition when environmental and socioeconomic features overlap. For healthcare practitioners, this means the model should be viewed as a screening tool rather than a final diagnostic authority. The accuracy gains reported in this study provide a robust foundation for prioritizing resources, but the ‘uncertainty’ at the boundary of severity levels underscores the need for localized clinical validation before implementing high-stakes medical interventions.

## Conclusion

The practical significance of this research lies in its potential to transition stunting screening from a purely descriptive approach to a predictive one. The hybrid feature augmentation architecture offers a robust framework for stunting prediction with a narrow margin of error. If integrated into existing health information systems with appropriate caution regarding its cross-sectional nature, it could serve as a valuable component of Ethiopia’s strategy to achieve the 2030 Sustainable Development Goals. Childhood stunting, a major problem in Ethiopia with a 35.4% prevalence, impairs development and leads to cognitive deficits, poor academic performance, increased disease risk, lower productivity, and chronic diseases. This study developed a hybrid machine learning model with explainable AI (XAI) to predict stunting in Ethiopian children under five and assess key risk factors. The model, leveraging Ethiopian Demographic and Health Survey (EDHS) data and combining ensemble and deep learning algorithms, achieved 94% accuracy, outperforming individual models. Key risk factors identified include child age, region, birth interval, and number of children under five in the household. Understanding these determinants is crucial for developing targeted interventions aimed at reducing stunting prevalence, which remains a significant public health challenge in Ethiopia. These findings align with existing literature and are crucial for targeted interventions. XAI techniques, particularly LIME, enhanced model transparency and fostered trust by providing insights into predictions. The hybrid model’s ability to provide accurate, explainable, and nuanced predictions of stunting, coupled with its identification of key risk factors, offers a powerful new approach to combating childhood malnutrition in Ethiopia. This study uses the 2019 Ethiopian Demographic and Health Survey (EDHS), which employs a robust stratified cluster sampling design. However, the cross-sectional nature of the data limits causal inference, only allowing for the identification of associations and predictive patterns. The 2019 data may also not reflect recent socio-political changes or global supply chain disruptions affecting nutritional equity in Ethiopia. Furthermore, recall bias is a concern, as many variables rely on maternal recall, potentially affecting the model’s precision. Future research should focus on validating these findings in larger, more diverse populations and exploring the model’s potential for integration into existing healthcare systems. Additionally, further investigation into the identified risk factors is warranted to understand the underlying mechanisms and develop more effective intervention strategies. The study serves as a strong foundation for future work aimed at translating AI-driven insights into tangible improvements in child health and nutrition outcomes. The high accuracy and robustness of the model also suggest its potential applicability to other areas of public health, such as predicting the risk of infectious diseases or identifying individuals at risk for chronic conditions.

## Data Availability

The data for this study come from the 2019 Ethiopian Demographic and Health Survey (EDHS), available through the Demographic and Health Surveys (DHS) Program website (https://dhsprogram.com/data/). While not publicly available, the data, which include demographic, socioeconomic, and health indicators, can be accessed upon registration, authorization, and reasonable request from the DHS Program. This allows researchers to conduct in-depth analyses that support evidence-based policymaking and program development.

## References

[1] Ayele, M. K., Baye, G. A. & Yesuf, S. H. Predicting stunting status among under five children in ethiopia using ensemblemachine learning algorithms. 1–11 (2025). 10.1038/s41598-025-03206-1 PMC12313921 40745172

[2] Soliman, A. et al. Early and long-term consequences of nutritional stunting: From childhood to adulthood. **92**, 1–12 (2021). 10.23750/abm.v92i1.11346 PMC7975963 33682846

[3] Ntawuyirushintege, S. et al. Spatiotemporal trends in stunting prevalence among children aged two years old in Rwanda (2020–2024): A retrospective analysis. 1–15 (2025). 10.3390/nu17172808 PMC12430221 40944196

[4] Sisay, Y. et al. Levels of stunting associated factors among under-five children in Ethiopia: A multi-level ordinal logistic regression analysis. 1–13 (2024). 10.1371/journal.pone.0296451 10.1371/journal.pone.0296451 PMC10760711 38165921

[5] Agushybana, F., Pratiwi, A., Kurnia, P. L. & Nandini, N. Reducing stunting prevalence: Causes, impacts, and strategies. 00009, 1–6 (2022).

[6] Suryana, E. A. The potential of economic loss due to stunting in the potential of economic loss due to stunting. *J. Ekon.* **8** (2023).

[7] Astika, T. et al. The accuracy of a novel stunting risk detection application based on nutrition and sanitation indicators in children aged under five years. (2025). 10.1186/s40795-025-01074-6 PMC12070535 40361193

[8] Doreswamy, D. & Nigus, M. Feature selection methods for household food insecurity classification. *Conf. Comput. Sci. Eng. Appl.* (ICCSEA, 2020). 10.1109/ICCSEA49143.2020.9132945

[9] Sarker, I. H. Machine learning: Algorithms, real-world applications and research directions. *SN Comput. Sci.* **2**, 1–21 (2021). 10.1007/s42979-021-00592-x PMC7983091 33778771

[10] Pintelas, P. & Livieris, I. E. Special issue on ensemble learning and applications. *Algorithms* **13**, (2020).

[11] Hasan, M. et al. Ensemble machine learning-based recommendation system for effective prediction of suitable agricultural crop cultivation. *Front. Plant. Sci.* **14**, 1–18 (2023). 10.3389/fpls.2023.1234555 PMC10449466 37636091

[12] Choudhary, K. et al. Recent advances and applications of deep learning methods in materials science. *Npj Comput. Mater* **8** (2022).

[13] Naskath, J., Sivakamasundari, G. & Begum, A. A. S. A Study on Different Deep Learning Algorithms Used in Deep Neural Nets: MLP SOM and DBN. *Wirel. Pers. Commun.* **128**, 2913–2936 (2023). 36276226 10.1007/s11277-022-10079-4 PMC9579606

[14] Deléglise, H. et al. Food security prediction from heterogeneous data combining machine and deep learning methods. *Expert Syst. Appl.* **190**, (2022).

[15] Mavaie, P., Holder, L. & Skinner, M. K. Hybrid deep learning approach to improve classification of low-volume high-dimensional data. *BMC Bioinform.* **24**, 1–20 (2023). 10.1186/s12859-023-05557-w PMC10631218 37936066

[16] Linardatos, P., Papastefanopoulos, V. & Kotsiantis, S. Explainable ai: A review of machine learning interpretability methods. *Entropy* **23**, 1–45 (2021). 10.3390/e23010018 PMC7824368 33375658

[17] Bao, A. & Zeng, Y. Understanding the dilemma of explainable artificial intelligence: a proposal for a ritual dialog framework. *Humanit. Soc. Sci. Commun.* **11**, 1–9 (2024).

[18] Analysis, D. *J. Artif. Intell.* **1**, 35–44 (2025).

[19] Fan, C., Chen, M., Wang, X., Wang, J. & Huang, B. A review on data preprocessing techniques toward efficient and reliable knowledge discovery from building operational data. *Front. Energy Res.* **9**, 1–17 (2021).

[20] Efficient data cleaning and preprocessing techniques for robust. Machine learning models efficient data cleaning and preprocessing.

[21] Brijith, A. Data Preprocessing for machine learning (2023).

[22] Brandt, B. J. & Lanzén, E. A. Comparative review of SMOTE and ADASYN in imbalanced data classification (2020).

[23] Motbainor, A., Arega, Z. & Tirfie, M. Comparing level of food insecurity between households with and without home gardening practices in Zege, Amhara region, North West Ethiopia: Community based study. *PLoS One*. **17**, 1–13 (2022). 10.1371/journal.pone.0279392 PMC9770380 36542650

[24] Chen, R. C., Dewi, C., Huang, S. W. & Caraka, R. E. Selecting critical features for data classification based on machine learning methods. *J. Big Data* **7**, (2020).

[25] Akhiat, Y. & Amjad, S. Feature selection: A review and comparative study feature selection: A review and comparative study. (2022). 10.1051/e3sconf/202235101046

[26] Liu, H., Wen, X., Polan, D. F., Brady, S. L. & Kaufman, R. A. An improved random forest algorithm an improved random forest algorithm (2020). 10.1088/1742-6596/1646/1/012070

[27] Cheng, X. A Comprehensive study of feature selection techniques in machine learning models. **1**, 1–14 (2024).

[28] Noelia, S. Filter methods for feature selection. A comparative study.

[29] Patel, D., Saxena, A. & Wang, J. A Machine learning-based wrapper method for feature selection. **20**, 1–33 (2024).

[30] Naseriparsa, M. A hybrid feature selection method to improve performance of a group of classification algorithms. **69**, 28–35 (2013).

[31] Pawluszek-Filipiak, K. & Borkowski, A. On the importance of train-test split ratio of datasets in automatic landslide detection by supervised classification. *Remote Sens.* **12**, (2020).

[32] Zhao, M. & Ye, N. High-dimensional ensemble learning classification: An ensemble learning classification algorithm based on high-dimensional feature space reconstruction. *Appl. Sci.* **14**, 1956 (2024).

[33] Li, Y., Wang, Z., Yang, A. & Yu, X. Integrating evolutionary algorithms and enhanced-YOLOv8 + for comprehensive apple ripeness prediction. 1–20 (2025). 10.1038/s41598-025-91939-4 PMC11873128 40025124

[34] Alshboul, O., Shehadeh, A., Almasabha, G. & Almuflih, A. S. Extreme gradient boosting-based machine learning approach for green building cost prediction. *Sustainability* **14**, (2022).

[35] Ampomah, E. K., Qin, Z. & Nyame, G. Evaluation of tree-based ensemble machine learning models in predicting stock price direction of movement (2020).

[36] Orrù, P. F. et al. Machine learning approach using MLP and SVM algorithms for the fault prediction of a centrifugal pump in the oil and gas industry. *Sustainability* **12**, (2020).

[37] Meena, G., Kumar, K., Kumar, S. & Lokesh, K. A hybrid deep learning approach for detecting sentiment polarities and knowledge graph representation on monkeypox tweets. *Decis. Anal. J.* **7**, 100243 (2023).

[38] Sipper, M. High per parameter: A large-scale study of hyperparameter tuning for machine learning algorithms. *Algorithms* **15** (2022).

[39] Ilemobayo, J. A., Durodola, O. I., Alade, O. & Awotunde, O. J. Hyperparameter tuning in machine learning: A comprehensive review (2024). 10.9734/jerr/2024/v26i61188

[40] Optimizing hyperparameters in machine learning models: Techniques and applications (2025).

[41] Steurer, M. et al. Based automated valuation models metrics for evaluating the performance of machine learning based automated valuation models. 0–16 (2021).

[42] Düntsch, I. & Gediga, G. Confusion matrices and rough set data analysis. *J Phys. Conf. Ser.* **1229**, (2019).

[43] Togunwa, T. O., Babatunde, A. O. & Abdullah, K. U. R. Deep hybrid model for maternal health risk classification in pregnancy: synergy of ANN and random forest. *Front Artif. Intell* **6**, (2023). 10.3389/frai.2023.1213436 PMC10354509 37476504

[44] Mehrpour, O. et al. Comparison of decision tree with common machine learning models for prediction of biguanide and sulfonylurea poisoning in the United States: an analysis of the National Poison Data System. *BMC Med. Inf. Decis. Mak.* **23**, 1–11 (2023). 10.1186/s12911-022-02095-y PMC10080923 37024869

[45] Zhang, Q. et al. *Landslide Susceptibility Mapp.* (2024).

[46] Plaia, A., Buscemi, S., Fürnkranz, J. & Mencía, E. L. Comparing boosting and bagging for decision trees of rankings. *J. Classif.* **39**, 78–99 (2022).

[47] Mokhtar, S. F., Sapiri, H., Studies, M. S. & Campus, S. P. Confidence Intervals Bootstrapping Approach: Significance Review. **19**, 30–42 (2023).

[48] Sokol, K. & Vogt, J. E. *What Does Evaluation of Explainable Artificial Intelligence Actually Tell Us? Extended Abstracts of the CHI Conference on Human Factors in Computing Systems (CHI EA ’24)*, May 11âfi16, 2024, Honolulu, HI, USA vol. 1 (Association for Computing Machinery, 2024).

